# Partial Deletion of eNOS Gene Causes Hyperinsulinemic State, Unbalance of Cardiac Insulin Signaling Pathways and Coronary Dysfunction Independently of High Fat Diet

**DOI:** 10.1371/journal.pone.0104156

**Published:** 2014-08-05

**Authors:** Cecilia Vecoli, Michela Novelli, Anna Pippa, Daniela Giacopelli, Pascale Beffy, Pellegrino Masiello, Antonio L’Abbate, Danilo Neglia

**Affiliations:** 1 Istituto di Fisiologia Clinica-CNR, Pisa, Italy; 2 Dipartimento di Ricerca Traslazionale e delle Nuove Tecnologie in Medicina e Chirurgia, Università di Pisa, Pisa, Italy; 3 Institute of Life Sciences, Scuola Superiore Sant’Anna, Pisa, Italy; 4 Fondazione Toscana G. Monasterio-CNR, Pisa, Italy; University of Illinois College of Medicine, United States of America

## Abstract

Abnormalities in eNOS gene, possibly interacting with high fat diet (HFD), affect peripheral vascular function and glucose metabolism. The relative role of eNOS gene, HFD and metabolic derangement on coronary function has not been fully elucidated. We test whether eNOS gene deficiency per se or in association with HFD modulates coronary function through mechanisms involving molecular pathways related to insulin signaling. Wild type (WT), eNOS^−/−^ and eNOS^+/−^ mice were studied. WT and eNOS^+/−^ mice were fed with either standard or HF diet for 16 weeks and compared with standard diet fed eNOS^−/−^. Glucose and insulin tolerance tests were performed during the last week of diet. Coronary resistance (CR) was measured at baseline and during infusions of acetylcholine (Ach) or sodium-nitroprusside (SNP) to evaluate endothelium-dependent or independent vasodilation, in the Langendorff isolated hearts. Cardiac expression of Akt and ERK genes as evaluation of two major insulin-regulated signaling pathways involved in the control of vascular tone were assessed by western blot. HFD-fed mice developed an overt diabetic state. Conversely, chow-fed genetically modified mice (in particular eNOS^−/−^) showed a metabolic pattern characterized by normoglycemia and hyperinsulinemia with a limited degree of insulin resistance. CR was significantly higher in animals with eNOS gene deletions than in WT, independently of diet. Percent decrease in CR, during Ach infusion, was significantly lower in both eNOS^−/−^ and eNOS^+/−^ mice than in WT, independently of diet. SNP reduced CR in all groups except eNOS^−/−^. The cardiac ERK1-2/Akt ratio, increased in animals with eNOS gene deletions compared with WT, independently of diet. These results suggest that the eNOS genetic deficiency, associated or not with HFD, has a relevant effect on coronary vascular function, possibly mediated by increase in blood insulin levels and unbalance in insulin-dependent signaling in coronary vessels, consistent with a shift towards a vasoconstrictive pattern.

## Introduction

Major risk factors for coronary artery disease (CAD) include a cluster of conditions such as obesity, dyslipidemia, hypertension and insulin resistance that is also referred to as metabolic syndrome. Unbalanced, fat-rich diet, which is a recognized cause of the metabolic syndrome, has been reported to have direct effects on vascular tone mainly by interfering with activation of eNOS [1], [2]. Thus, abnormal endothelial function is a common feature of the syndrome and is usually considered to be secondary to the metabolic abnormalities.

More recently, it has been hypothesized that endothelial dysfunction may precede or even cause metabolic deregulation. Nitric oxide (NO), a major factor in the regulation of vascular function, is believed to also exert a significant role in the maintenance of glucose homeostasis, by contributing to modulation of peripheral insulin sensitivity [3], [4] and possibly insulin secretion [5], [6]. Both arterial hypertension and insulin resistance have been documented in either endothelial NO synthase (eNOS) null mice [7], [8], [9] or mice with partial deletion of the eNOS gene when challenged with a nutritional stress such as high fat diet (HFD) [10]. In the clinical setting, polymorphic variants of the eNOS gene are associated with arterial hypertension and insulin resistance in various populations [11]–[13]. In particular, in a recent paper from our group, we showed that the CC polymorphic variant of eNOS −786 T/C polymorphism was associated with a significant increase in arterial blood pressure and impairment of glucose metabolism in a population of cardiomyopathic patients [13]. These recent experimental and clinical evidences suggest that eNOS gene abnormalities, possibly interacting with HFD, may contribute to endothelial/vascular dysfunction as well as to deregulation of glucose metabolism. Whether eNOS genetic determinants and metabolic abnormalities might interact to affect coronary circulatory function has not been fully elucidated.

In the present study, we measured coronary function in eNOS^−/−^ mice as well as in eNOS^+/−^ partially deficient mice, with or without HFD. Specifically, baseline coronary resistance and coronary response to vasodilating stimuli were measured in isolated hearts from total or partial eNOS knockout mice fed or not with HFD for 16 weeks, after in vivo assessment of glucose tolerance and insulin sensitivity. In addition we tested whether eNOS gene deficiency directly modifies insulin-dependent cardiac molecular pathways involved in the control of coronary tone. NOS expression, insulin-dependent triggering of PI3K/Akt pathway, resulting in eNOS phosphorylation and activation, as well as stimulation of (MAPK)/ERK pathway, that leads to production of the vasoconstrictor factor endothelin (ET)-1 [14], [15], were evaluated in the cardiac tissue in the same groups.

## Methods

Experiments were carried out in wild-type (WT) C57BL/6 mice and in mice with B6/129 eNOS^−/−^ genotype backcrossed onto the C57BL/6 strain [8], [16]. C57-Bl/6J female wild type (WT) and male eNOS^−/−^ mice were mated to generate eNOS^+/−^ mice. To assess genotype, genomic DNA was extracted from mouse tail biopsies and genotyped by polymerase chain reaction (PCR) according to a protocol provided by the Jackson Laboratory [http://www.jax.org]. For this study, male mice only were included in the experimental groups (WT, eNOS^−/−^ and eNOS^+/−^). Starting at the age of 4 weeks, WT and eNOS^+/−^ mice were fed for 16 weeks either a standard diet (chow food, containing protein 23%, fat 5%, carbohydrate approximately 60%, fiber 5% and ash 5.5%, with 10% energy deriving from fat), or a high-fat diet (HFD) containing protein 23%, fat 34%, carbohydrate approximately 30%, fiber 5% and ash 5.5%, with 60% energy deriving from fat. Both diets were purchased from Mucedola, Settimo Milanese, MI, Italy). eNOS^−/−^mice, being per se insulin resistant and hypertensive, were nursed only with standard diet. Therefore, five groups of animals were studied: 1) WT (n = 16); 2) HFD-fed WT (n = 18); 3) eNOS^+/−^ (n = 15); 4) HFD-fed eNOS^+/−^ (n = 16) and 5) eNOS^−/−^ (n = 10).

Body weight and blood glucose levels (measured by a glucometer, Glucocard GT-1610, Menarini Diagnostic, Firenze, Italy) were measured weekly in animals left without food 4 h before testing.

Experimental protocols were approved by the Animal Care Committee of the Italian Ministry of Health and conformed to the “Guiding Principles for Research Involving Animals and Human Beings” approved by the Council of the American Physiological Society. Throughout the study period, the mice were housed under controlled 12/12 h light/dark cycle and given food and water ad libitum. To avoid oxidation and organoleptic alteration of HFD, this food was added fresh every other day.

### Intraperitoneal glucose tolerance test

After 15 weeks of diet, glucose (1.5 g/kg body weight as 16.5% solution) was given intraperitoneally to 4-h-fasting conscious animals. Drops of blood taken from the tail vein before and 15, 60, 120 and 180 min after the glucose injection were immediately used for glucose determination by a glucometer. Some blood samples (at 0, 15 and 60 min) were also collected in EDTA-treated tubes and centrifuged at 4°C; plasma was stored at −20°C for subsequent insulin measurement. Insulin was determined by radioimmunoassay (RIA), according to Herbert et al. [17], using anti-insulin antibody and ^125^I-labeled insulin obtained from Linco (Linco Research, INC. St. Charles, MO, USA).

### Intraperitoneal insulin tolerance test

After 16 weeks of diet, human insulin (0.75 U/kg body weight, Humulin R, Eli Lilly, Indianapolis, IN, USA) was administered to conscious animals by intraperitoneal route. Blood glucose was measured by a glucometer in drops of blood taken from the tail vein before and 15, 30, 60 and 120 min after insulin administration.

### Isolated heart preparation

At the end of the experiment, after 16 weeks of diet, at least 6 mice of each group were anaesthetized with pentobarbital i.p. injection and heparinized via the left femoral vein (500 U i.m.). The heart was rapidly excised, weighed and placed in cold perfusion medium. The isolated hearts were immediately attached to the Langendorff apparatus and retrogradely perfused at constant pressure (at 37°C), as previously described [18]. The perfusion medium consisted of oxygenated Krebs-Henseleit buffer (NaCl 118.5; NaHCO_3_ 25.0; KCl 4.7; MgSO_4_ 1.2; KH_2_PO_4_ 1.2; glucose 11.0; CaCl_2_ 1.4 mmol/L) and gassed with 95% O_2_ and 5% CO_2_ (pH 7.4). Coronary flow was continuously monitored while collecting the cardiac effluent. Coronary resistance (CR) was measured as input pressure divided by coronary flow per gram of myocardial tissue (mmHg*min*g/mL).

Isolated hearts were studied according to one of two protocols: after a stabilization period (30 min, baseline), the heart was perfused either with acetylcholine (Ach, 10 µmol/L) to measure the degree of endothelium-dependent vasodilation (at least 6 mice for each group) or with nitroprusside (SNP, 10 µmol/L) to measure the degree of endothelium-independent vasodilation (at least 6 mice for each group). Ach and SNP were infused into the aortic cannula for 3 minutes at infusion rates ranging from 0.2% to 1% of coronary flow. Injection of vehicle alone did not cause changes in coronary flow. The concentrations of Ach and SNP were selected on the basis of pilot experiments aimed at assessing the concentrations giving the maximum degree of vasodilation.

### Western blot analysis of cardiac tissue for Akt, pAKT, ERK1-2, pERK1-2, eNOS, peNOS

From at least four randomly chosen animals in each group, hearts were immediately frozen in liquid nitrogen and subsequently pulverized. Lysates were prepared using lysis buffer (50 mM Tris HCl pH 7.5, 150 mM NaCl, 1 mM EDTA, 1% Triton X-100, 1 mM PMSF, 2 mM Na_3_VO_4_, 2 mM NaF), containing Protease Inhibitor Cocktail. Protein concentration in supernatant was determined using the Bio-Rad protein assay (Bio-Rad Laboratories, Hercules, CA, USA). Equal amounts of protein (30 µg of heart tissue lysate) were submitted to 10% SDS/polyacrylamide gel electrophoresis and transferred to a nitrocellulose membrane. The membranes were blocked and then incubated overnight al 4°C with the primary antibody, followed by an appropriate secondary antibody. Secondary antibody binding was visualized using the ECL detection kit (Euroclone, Milan, Italy), according to the manufacturer’s instructions. Densitometric analysis of developed blots was performed with the ImmageJ program. Protein bands were quantified and values were normalized to those of α-tubulin (Sigma, St. Louis, MO, USA). Antibodies to Akt and pAkt were purchased from Cell Signaling Technology (Beverly, MA, USA); antibodies to eNOS, peNOS, ERK1-2, and pERK1-2 were purchased from Santa Cruz Biotechnologies (Santa Cruz, CA, USA). The pERK/pAkt ratio was calculated as double ratio between phosphorylated and not phosphorylated form of ERK1-2 (pERK1-2/ERK1-2) and phosphorylated and not phosphorylated form of Akt (pAkt/Akt).

### RT-PCR analysis of cardiac tissue for iNOS

The mRNA expression levels of iNOS were determined by semi-quantitative PCR method. RNA was extracted from myocardial tissue using the RNeasy Mini Kit (Qiagen, Milan, Italy). RNA was quantified by measuring absorbance at 260 nm and stored at –80°C until use. One microgram of total RNA was reversed transcribed using iScriptTM cDNA Synthesis Kit (Bio-Rad, Milan, Italy). Quantification of mRNA for these PCR products was accomplished by using GoTaqGreen Master Mix Kit (Promega, Madison, WI) with the Applied Biosystems 2720 Thermal Cycler (Life Technologies, Foser City, CA). The forward and reverse primers were purchased from QIAGEN (QuantiTect Primer Assay). The PCR products were then subjected to agarose gel electrophoresis (1%), pre-stained with GelRedTM (Biotium, Hayward, CA), and photographed. Data analysis were performed using ImmageJ program. PCR results were normalized to ribosomal gene Rn18S as housekeeping gene.

### Statistical analysis

Results are presented as mean ± SEM of the number (n) of replicate determinations. Statistical significance between different experimental groups was determined by using a two-way ANOVA followed by the Fisher’s exact test as post-hoc test. A p<0.05 was considered significant.

## Results

### Body weight and basal glycemic levels

**Figure 1:**
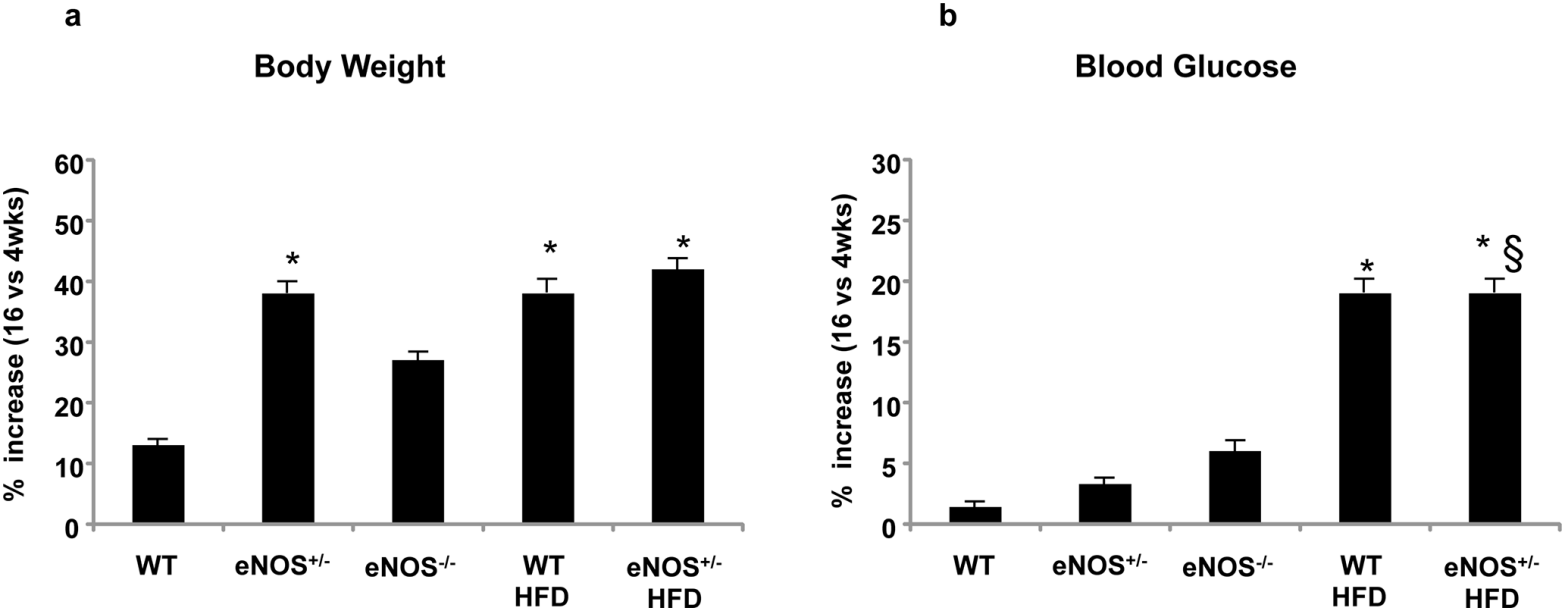
Percent increase (16 versus 4 weeks) of body weight (a) and blood glucose levels (b) during the 16 weeks of monitoring in all groups of animals, indicated as follows: WT, healthy controls fed with standard diet; eNOS^+/−^, partial knockout eNOS mice fed with standard diet; eNOS^−/−^, total knockout eNOS mice fed with standard diet; WT HFD, healthy controls fed with high-fat diet (HFD); eNOS^+/−^ HFD, partial knockout eNOS mice fed with HFD. Mean ± SEM of 15–18 mice for each group, except eNOS^−/−^ mice (n = 10). *p<0.05, at least, *vs* WT; §p<0.05, at least, *vs* eNOS^+/−^.

During the experimental period, body weight increased progressively in all groups of animals, with some differences in the growth rate and final weight attained at the end of the 16 weeks of diet (Table 1). As expected, HFD-fed mice gained significantly higher body weight than corresponding standard diet groups. The percent increase in body weight from 4 to 16 weeks of diet was significantly higher not only in HFD-fed mice but also in eNOS^+/−^, as compared with WT group (Fig. 1a). No differences in the daily amount of food and water intake were observed (not shown). In chow-fed WT, eNOS^+/−^ and eNOS^−/−^ mice, basal blood glucose levels changed very little during the 16 weeks of observation, whereas in both the HFD-fed groups they were significantly higher than those of controls already after 4 weeks of diet and progressively increased thereafter (Table 1 and Fig. 1b).

**Table 1:** Body weight and basal blood glucose levels during the 16 weeks of controlled diet in all groups of animals.

	4 wks	8 wks	12 wks	16 wks
***Body Weight (g)***				
**WT**	25.8±1.1	27.8±1.4	28.±1.0	29.2±0.9
**eNOS^+/−^**	23.3±0.9	26.9±1.0	30.6±1.2	32.3±0.8
**eNOS^−/−^**	26.0±1.8	28.1±1.5	31.4±1.0	32.7±1.7
**WT HFD**	26.3±0.9	32.0±1.2*	35.1±1.3*	36.4±0.6*
**eNOS^+/−^ HFD**	24.5±1.5	29.0±1.2* §	32.4±0.9* §	34.9±1.3* §
***Blood Glucose (mg/dL)***				
**WT**	144±5	148±5	147±4	146±4
**eNOS^+/−^**	148±5	151±5	150±5*	153±4*
**eNOS^−/−^**	145±8	152±10	148±7	154±6*
**WT HFD**	160±10*	168±10*	179±15*	190±9*
**eNOS^+/−^ HFD**	160±8* §	174±9* §	185±5* §	192±8* §

WT: wild type controls HFD: high fat diet.

*p<0.05, at least, vs WT;

§ p<0.05, at least, vs eNOS^+/−^.

Just before starting diet regimens (at 4 weeks of age), body weights of animals’ groups were: WT, 22.1±0.9 g; eNOS^+/−^, 21.7±1.2 g; eNOS^−/−^, 22.0±1.3 g; blood glucose levels were: WT, 142±4 mg/dL; eNOS^+/−^, 138±9 mg/dL; eNOS^−/−^, 140±8 mg/dL.

### Glucose tolerance and insulin sensitivity

**Figure 2:**
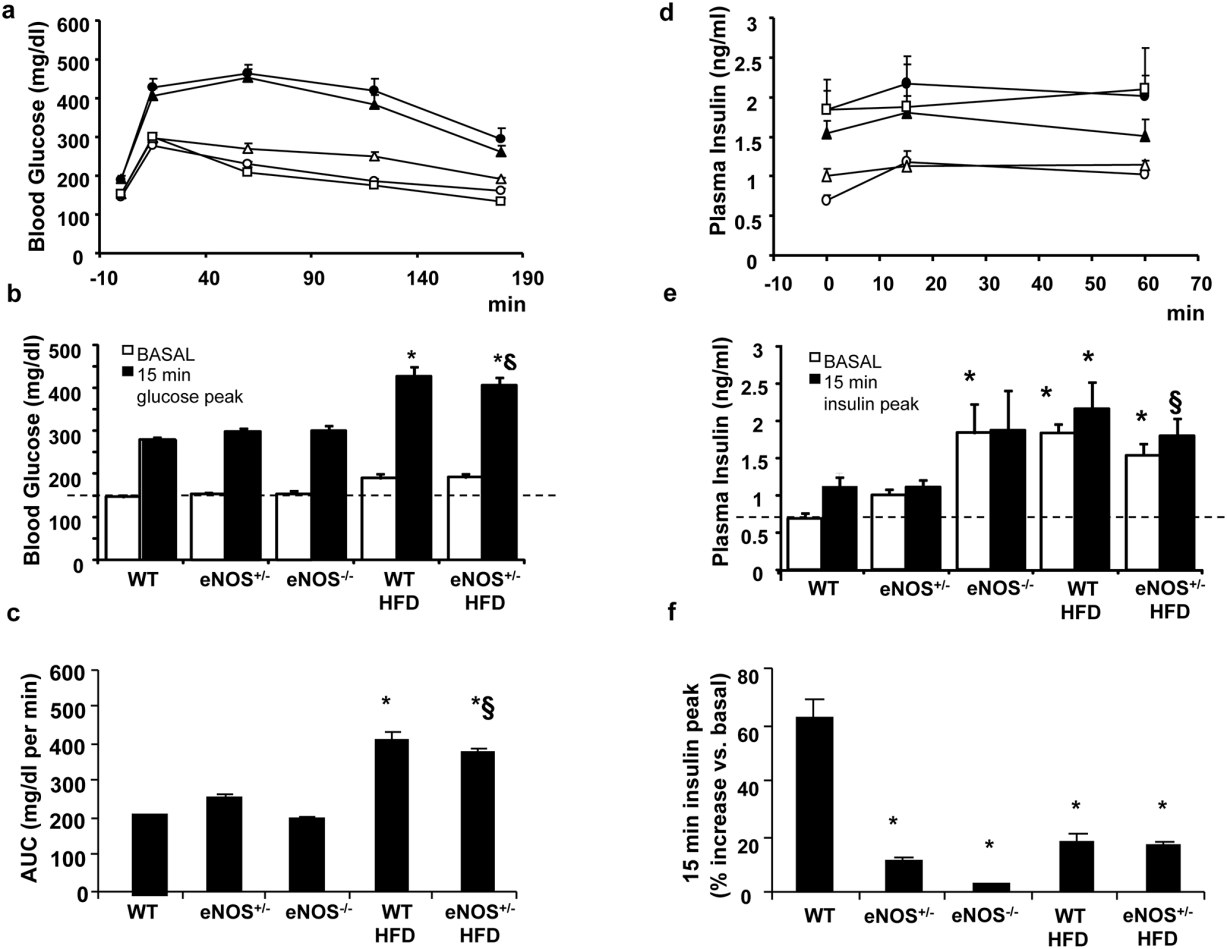
Blood glucose and plasma insulin levels during glucose tolerance test. Abbreviations are defined in Fig. 1. a) Blood glucose levels were determined during a glucose tolerance test (glucose 1.5 g/kg i.p.) performed after 15 weeks of HFD diet. b) values of blood glucose at baseline and 15 minutes after the glucose load. c) the area under the curve (AUC) for glycaemic values. d) Plasma insulin levels were determined during a glucose tolerance test (glucose 1.5 g/kg i.p.) performed after 15 weeks of HFD diet. e) Plasma insulin levels at baseline and 15 minutes after the glucose load (insulin peak). f) % increase of 15-min post-loading plasma insulin level with respect to the corresponding basal value. Abbreviations are as in Fig. 1. In the panels a) and d) empty circles are WT mice, empty triangles are eNOS^+/−^ mice, empty squares are eNOS^−/−^ mice, filled circles are WT HFD mice, and filled triangles are eNOS^+/−^ HFD. Mean ± SEM of 15–18 mice for each group, except for eNOS^−/−^ mice (n = 4). *p<0.05, at least, vs WT; §p<0.05, at least, vs eNOS^+/−^.

The intraperitoneal glucose tolerance test revealed that both groups of animals fed with HFD (WT and eNOS^+/−^ mice), which already had significantly higher basal glucose levels than controls, were clearly glucose intolerant (Fig. 2a). Indeed, they had the highest post-loading glucose peaks at 15 minutes (Fig. 2b) and the largest areas under the curve (Fig. 2c) as compared to the other groups. The corresponding post-loading insulin profile (Fig. 2d) showed that the HFD-fed mice, which had significantly higher basal insulin levels than chow-fed WT, slightly augmented these levels at 15 and 60 min after the glucose load, although the percent increase over basal at the 15 min peak was limited as compared to controls (Fig. 2e). Interestingly, despite normoglycemia, eNOS^−/−^ mice had increased insulin levels at baseline, that remained high after the load, without any further increase in response to glucose (Fig. 2f).

**Figure 3:**
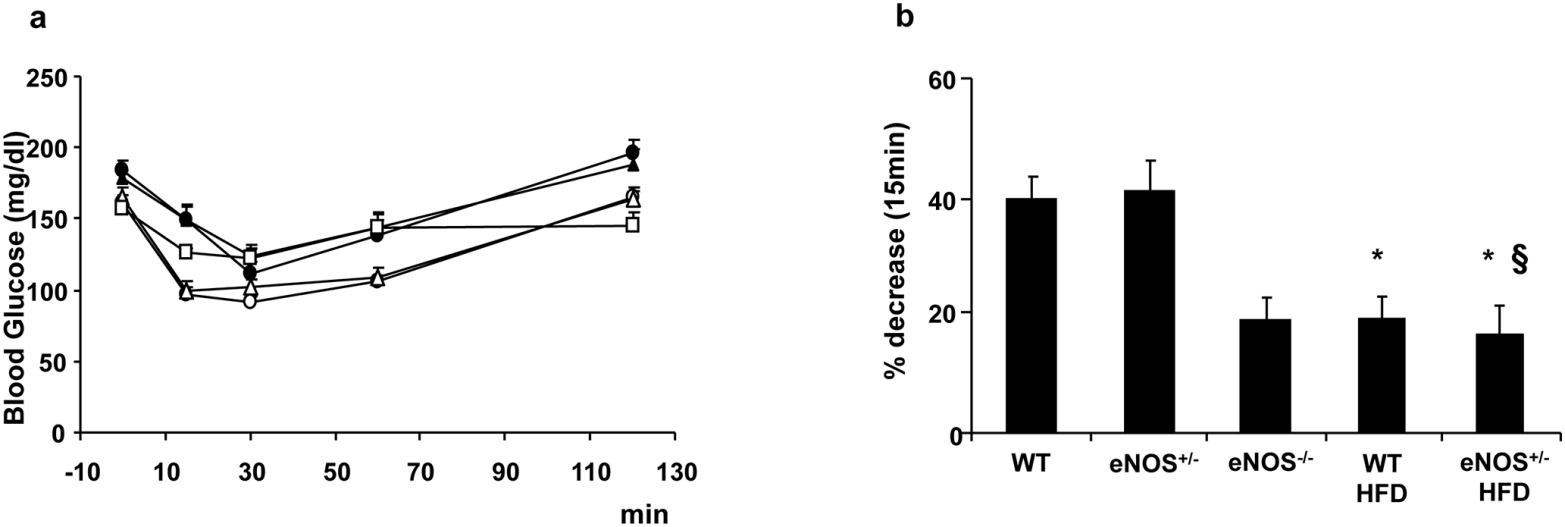
Blood glucose levels during insulin tolerance test. Abbreviations are as in Fig. 1. a) Blood glucose levels were determined during an insulin tolerance test (insulin 0.75 U/kg i.p.) performed after 16 weeks of diet. b) percent decrements of blood glucose values with respect to basal at 15 minutes after the insulin administration. Abbreviations are as in Fig. 1. Empty circles are WT mice, empty triangles are eNOS^+/−^ mice, empty squares are eNOS^−/−^ mice, filled circles are WT HFD mice, and filled triangles are eNOS^+/−^ HFD Mean ± SEM of 15–18 mice for each group, except for eNOS^−/−^ mice (n = 4). *p<0.05, at least, *vs* WT; §p<0.05, at least, *vs* eNOS^+/−^.

An intraperitoneal insulin tolerance test (Fig. 3) showed that the HFD-fed groups were insulin resistant, as indicated by a significantly lower glucose clearance from the bloodstream in response to administered insulin.

In chow-fed eNOS^+/−^ mice the hypoglycaemic effects of exogenous insulin administration were super-imposable to those of controls, while eNOS^−/−^ mice showed a different trend, as the early limited reduction of blood glucose (Fig. 3b) and the subsequent glycemic values until 60 min were closer to HFD-fed animals than to controls (Fig. 3a).

### Coronary vascular resistance

**Figure 4:**
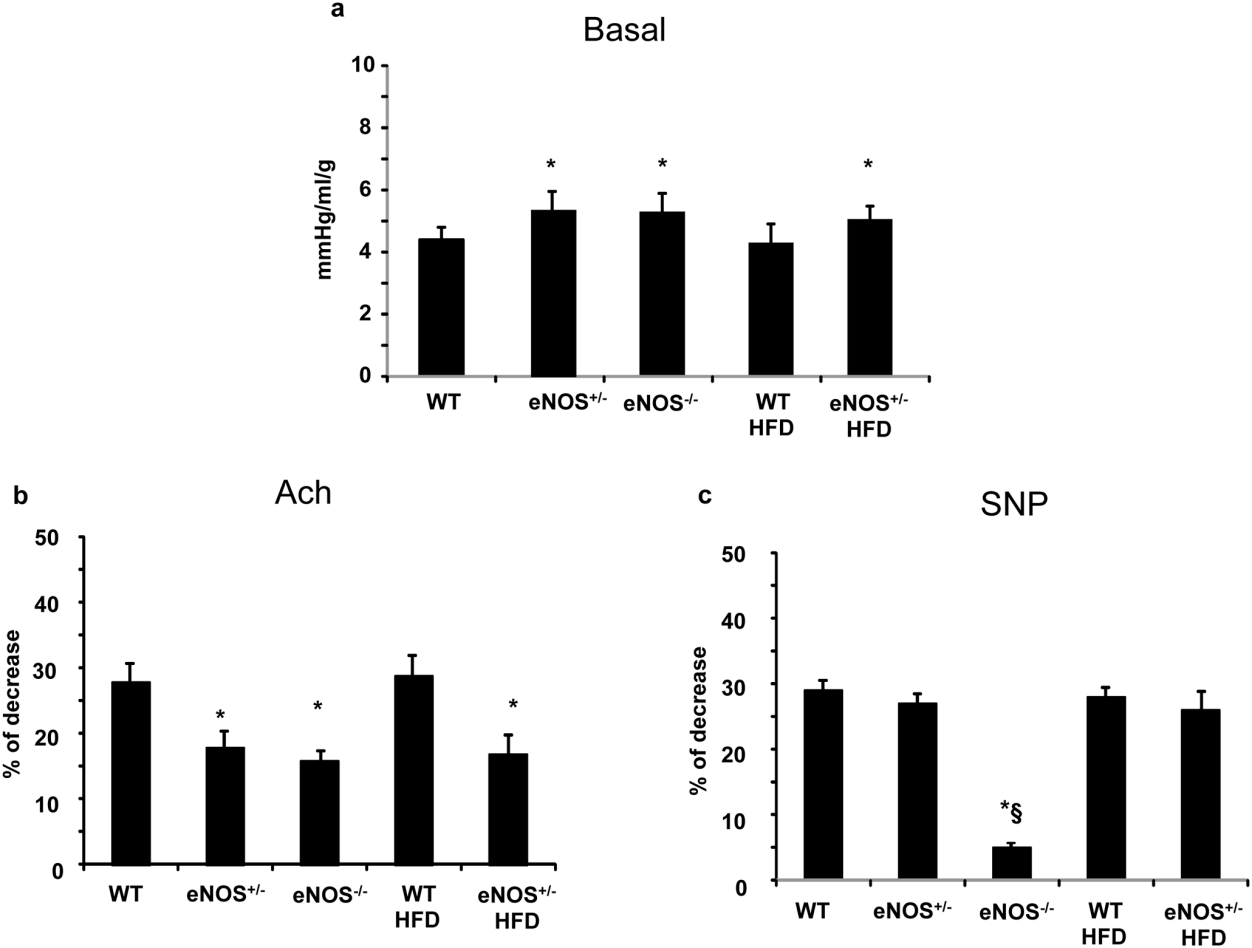
Coronary resistance (CR) in Langendorff configuration in basal conditions (a); b) Percent change in CR during 3 minutes of acetylcholine (Ach) infusion with respect to the corresponding basal value; c) percent change in CR during 3 minutes of sodium nitroprusside (SNP) infusion. Abbreviations are as in Fig. 1. Mean ± SEM of at least 6 mice for each group. *p<0.05, at least, *vs* WT; §p<0.05, at least *vs* eNOS^+/−^.

Coronary vascular resistance (CR) was significantly higher in animals with eNOS gene deletions than in WT mice, independently of diet (Fig. 4a). In particular, as compared with WT, CR was increased by 21%, 20% and 14% in eNOS^+/−^, eNOS^−/−^ and HFD-fed eNOS^+/−^, respectively. Percent decrease in CR, during endothelium-dependent vasodilating stimulus by Ach, was significantly lower in eNOS genetically modified mice than in WT, again independently of diet (p<0.05) (Fig. 4b). Conversely, the endothelium-independent vasodilator SNP was effective in all groups of animals except eNOS^−/−^. This latter group showed negligible reduction in CR after SNP (∼5% decrement), suggesting the occurrence of irreversible coronary vascular abnormalities (Fig. 4c).

### NOS expression in the heart

**Figure 5:**
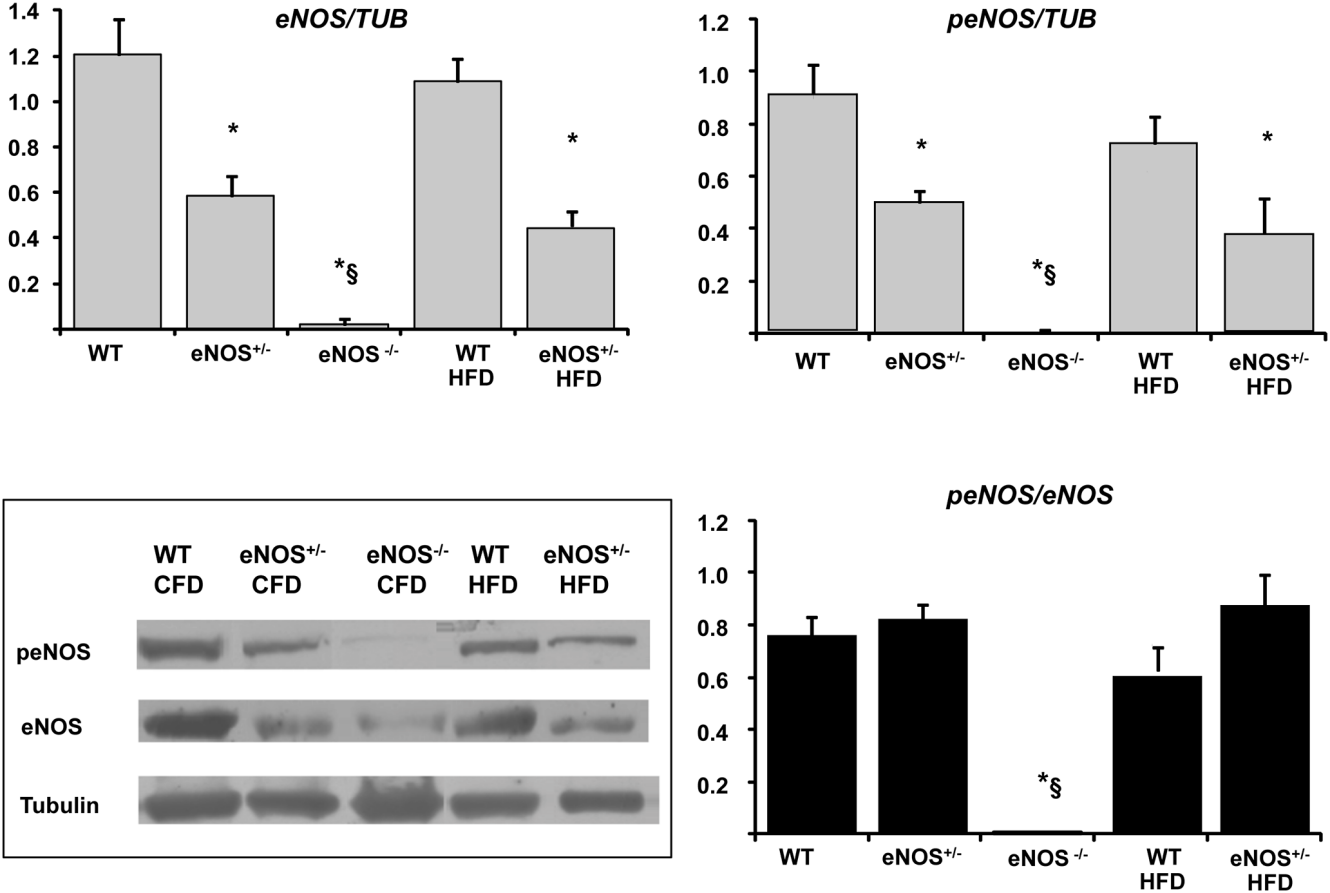
eNOS expression in cardiac tissue of experimental animals. Cardiac tissue homogenates were subjected to Western blotting for eNOS, peNOS and α-tubulin protein determination. Representative immunoblots (inferior left panel) are shown for all groups of mice. Abbreviations are defined in Fig. 1. Mean ± SEM of 4–6 mice for each group. *p<0.05, at least, *vs* WT; §p<0.05, at least, *vs* eNOS^+/−^.

**Figure 6:**
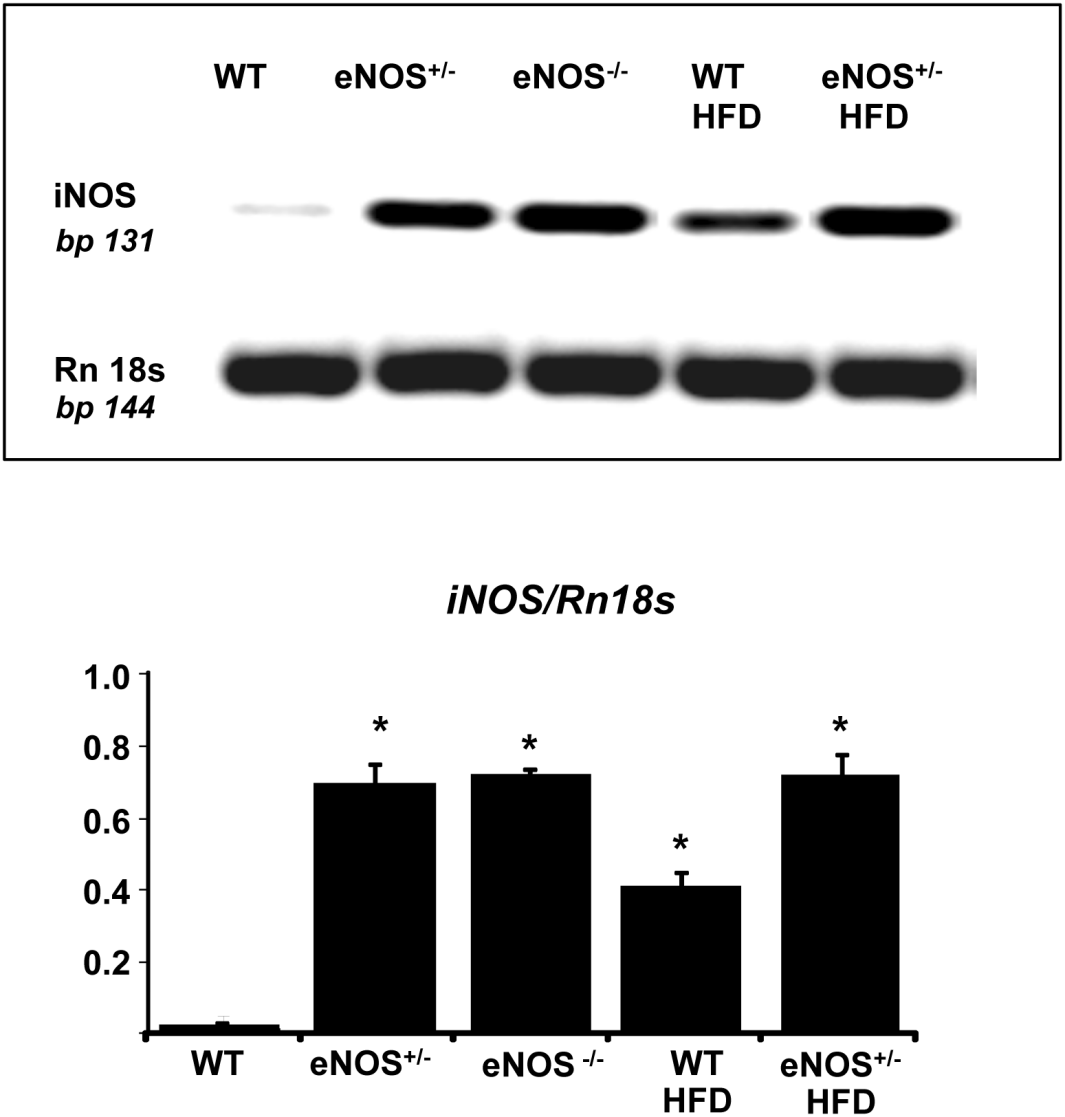
iNOS gene expression in cardiac tissue of experimental animals. Cardiac tissue homogenates were subjected to RT-PCR. Results were normalized to ribosomal gene Rn18S as housekeeping gene. Abbreviations are defined in Fig. 1. Mean ± SEM of 4–6 mice for each group. *p<0.05, at least, *vs* WT.

As expected, western blot analysis documented that eNOS protein expression was reduced by approximately a half in eNOS^+/−^ mice compared with WT and was absent in eNOS^−/−^ (Fig. 5). HFD did not alter eNOS expression. The ratio peNOS/eNOS was affected neither by partial eNOS deletion nor by HFD. Inducible NOS isoform (iNOS) was over-expressed in all groups with respect to WT controls (Fig. 6). The up-regulation of iNOS was more evident in the eNOS genetically modified animals, but HFD per se (in WT mice) was also able to moderately increase the levels of this enzyme.

### ERK and Akt signaling pathways in the heart

**Figure 7:**
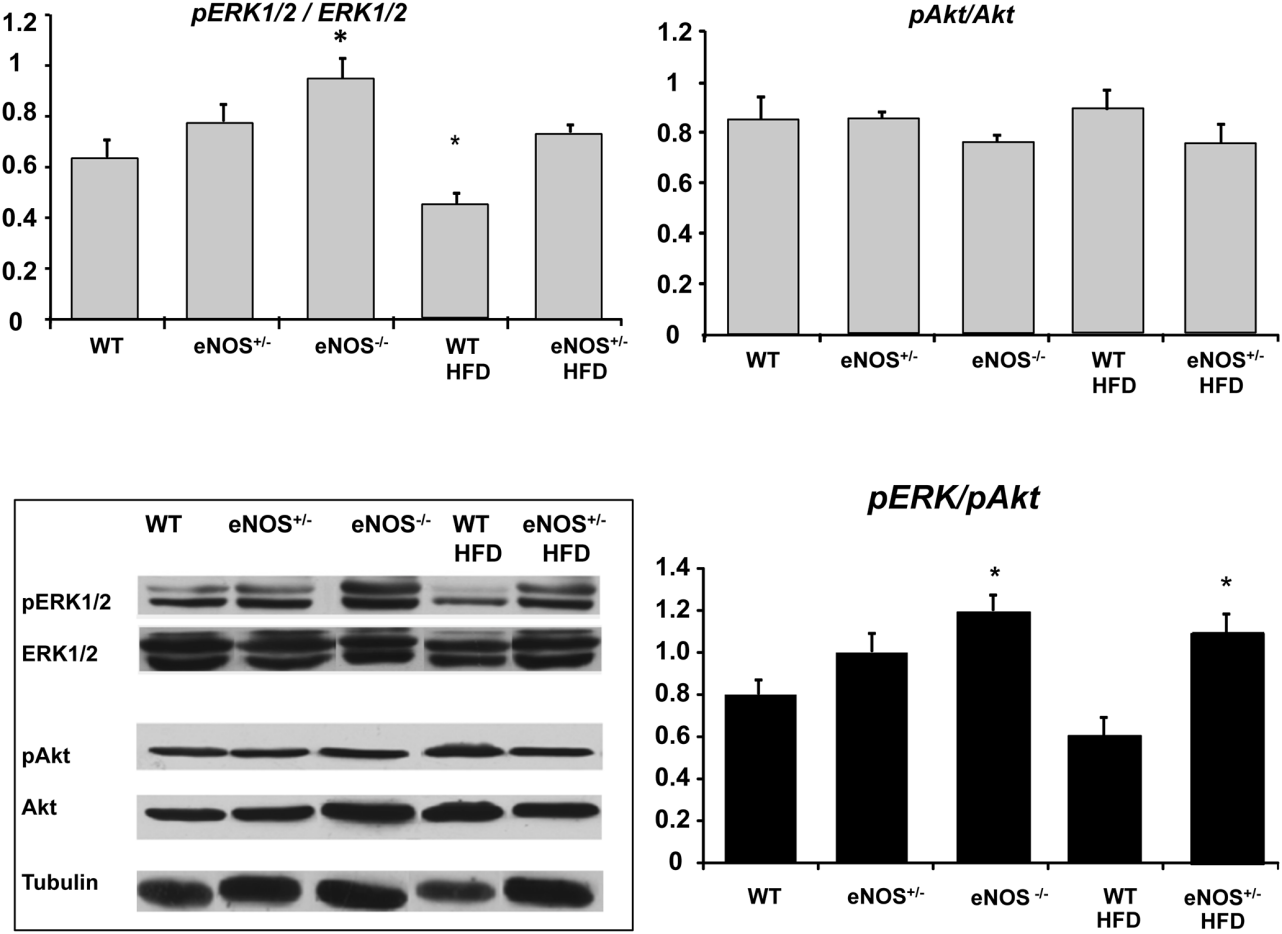
Expression of ERK1-2 and Akt in cardiac tissue of experimental animals. Cardiac tissue homogenates were subjected to Western blotting for Akt, pAkt, ERK1-2, and pERK1-2 protein determination. Representative immunoblots (inferior left panel) are shown for all groups of mice. Abbreviations are defined as in Fig. 1. Histograms showing the double pERK/p-Akt ratio have been reported. Mean ± SEM of 4–6 mice for each group. *p<0.05, at least, *vs* WT; §p<0.05, at least *vs* eNOS^+/−^.

eNOS genetic abnormalities were associated with a moderate activation of ERK1-2 pathway (as implied by a tendency to an increased pERK/ERK1-2 ratio, that attained statistical significance in the eNOS^−/−^ group), concomitantly with a small deactivation of the Akt pathway, as indicated by a slight decrease in the pAkt/Akt ratio (Fig. 7). As a result, the double ratio of pERK/pAkt showed a clear trend to increase in all eNOS-genetically modified groups as compared with WT (statistical significant in eNOS^−/−^ and HFD-fed eNOS^+/−^), pointing out a definite imbalance between the two molecular pathways downstream the insulin receptor. HFD did not modify the trend in eNOS^+/−^ group, while moderately decreasing the pERK/pAkt ratio mainly due to a significant reduction in the pERK/ERK1-2 ratio.

## Discussion

**Figure 8:**
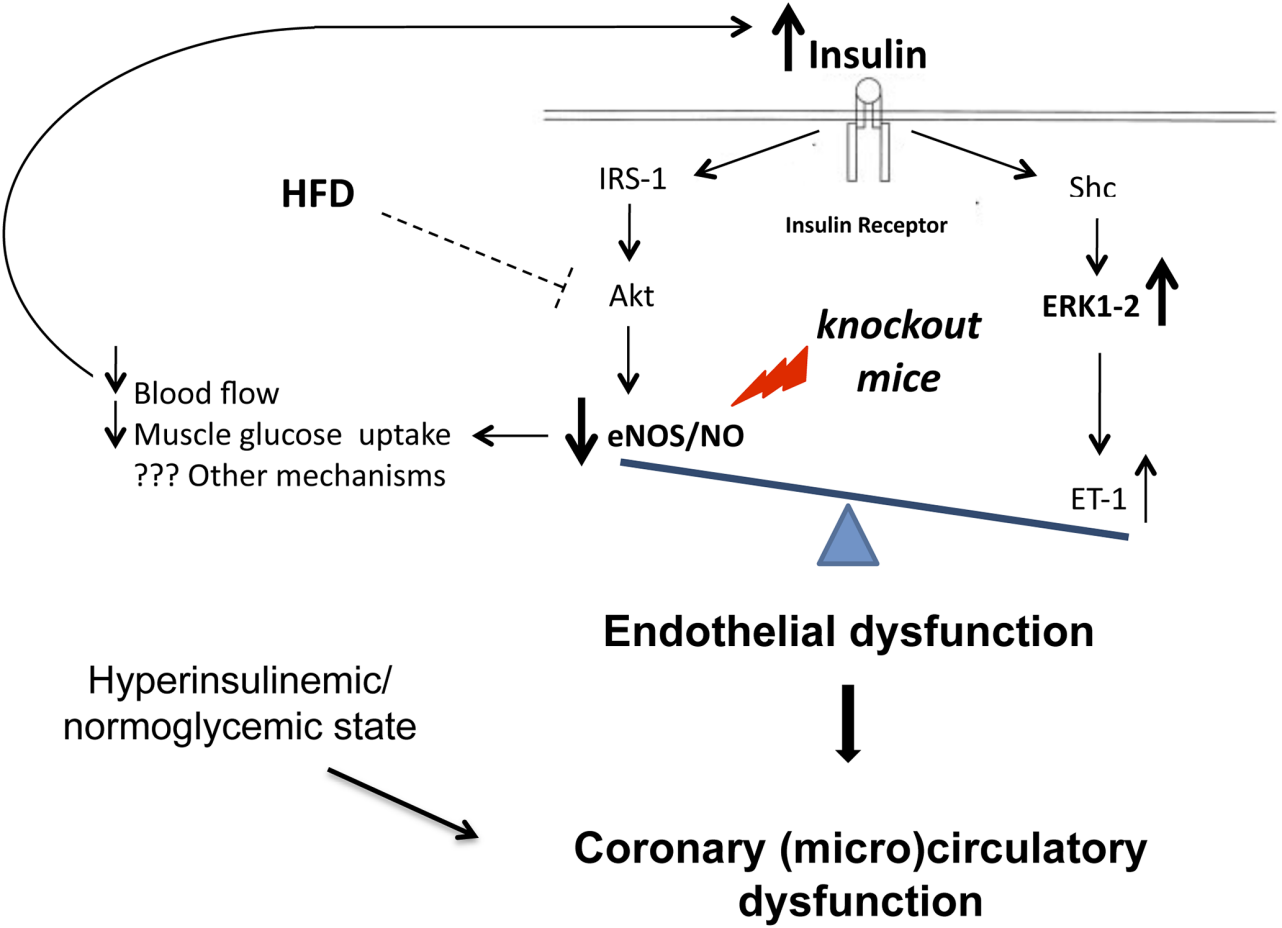
Diagrammatic representation of proposed mechanisms of interaction between eNOS deficiency, metabolic stress and insulin-dependent signaling pathway in coronary endothelial cells.

In the present experimental study, deletion of eNOS gene, either partial or total, results in a significant impairment in coronary vasodilating capability. Genetically modified mice also show a peculiar abnormality of glucose homeostasis characterized by a normoglycemic/hyperinsulinemic state with partial preservation of peripheral insulin sensitivity. In the myocardium of these animals, the molecular pathways downstream the insulin receptor are altered leading to a shift of the ERK1-2/Akt balance towards a prevalent vasoconstrictive pattern. HFD, while causing overt diabetes and marked insulin resistance, does not independently affect coronary tone and the balance of cardiac molecular pathways downstream insulin receptor. A diagrammatic representation of proposed mechanisms of interaction between eNOS deficiency, metabolic stress and insulin- dependent signaling pathways in endothelial cells is shown in Figure 8.

### Effects of eNOS gene deletions and HFD on coronary vascular tone

Our findings demonstrate that partial or total deletion of eNOS gene is able per se to impair coronary vascular tone. Gödecke et al [19] firstly studied coronary hemodynamics in eNOS^−/−^ mice by Langendorff-perfused hearts, showing no changes in basal coronary flow. Possible differences in animal models could account for such discrepancy. Indeed, in our study, eNOS deficiency was associated with increased coronary resistance both at baseline and during Ach infusion as compared with WT and HFD-WT groups. Both eNOS^−/−^ and eNOS^+/−^ had the same impaired response to endothelium-dependent vasodilation, suggesting that NO bioavailability is insufficient when 50% of the enzyme is lacking. Interestingly, the endothelium-independent vasodilation capability (assessed by SNP infusion) was still preserved in eNOS^+/−^, whereas it was totally blunted in eNOS^−/−^. It can be hypothesized that in total knockout mice, severe eNOS gene abnormalities affect coronary function with additional mechanisms besides the impaired endothelium dependent vasodilating capability. Structural alterations of the coronary arterioles are most likely involved, as observed in the systemic microvasculature of adult hypertensive eNOS^−/−^mice [20], [21].

In the present study, HFD caused a somewhat expected overt diabetic state concomitantly with basal hyperinsulinemia, indicative of insufficient compensation of a quite relevant insulin resistance, but such metabolic alteration was neither associated per se with abnormal coronary vascular function nor induced synergistic changes at coronary level when used in eNOS-deficient animals. In previous studies performed in partially eNOS-deficient mice subjected to HFD, the two conditions were additive in causing an increase in systemic vascular resistance and hypertension [10]. It is conceivable that different vascular districts may have different behaviors. Moreover, while systemic resistance is measured in vivo, coronary resistance can be only measured ex-vivo, in the absence of the in vivo circulating milieu, which in turn may differently affect vascular function.

In genetic (db/db mice) or chemically-induced animal models of diabetes with marked hypoinsulinemia, we have previously shown the occurrence of increased coronary resistance ex-vivo, associated with decreased cardiac expression of eNOS [18], [22], [23]. In the same models, pharmacological treatments able to restore the eNOS expression were also able to reduce coronary resistance, leading to improvement of cardiac function [18], [23]. Differences in the animal models employed may account for the apparent difference between previous and present results. In particular, in the genetic model of db/db mice, diabetes and obesity are the result of a deficit in the leptin receptor, while the beta-cytotoxic agent streptozotocin causes a severe hyperglycemic/hypoinsulinemic state. In the present experiments, diabetes associated with insulin resistance and partially compensatory hyperinsulinemia was induced by HFD; however, HFD per se was unable to interfere with coronary resistance.

### Effects of eNOS gene deletions on insulin/glucose homeostasis

Both partial and total deletion of the eNOS gene were able to affect insulin/glucose homeostasis causing hyperinsulinemia but not the overt insulin resistant state induced by HFD. Indeed, mainly the total deletion of eNOS gene (the partial deletion had a minor effect) was associated with increased circulating insulin levels in the basal state and during the glucose tolerance test, in the absence of basal hyperglycemia or glucose intolerance. This metabolic impairment is somewhat different from the clear-cut insulin resistant state produced by HFD, as the high circulating levels of insulin in eNOS-deficient animals maintained normoglycemia in basal condition and allowed a normal glucose clearance during glucose tolerance test, despite the lack of a definite post-loading insulin peak.

A generic insulin resistant state has been already described in eNOS^−/−^ [7], [24] and in eNOS^+/−^ mice fed with HFD [10], [25]. The exact mechanism by which eNOS gene deficiencies lead to increased circulating insulin awaits further investigations, although several authors suggest that the increased peripheral vascular resistance and the consequent reduction of blood flow might reduce glucose uptake by skeletal muscle, thereby favouring a trend to increased blood glucose levels which on turn stimulate insulin secretion [7], [10], [24] [25]. This is also interpreted as a compensatory mechanism that in physiological/healthy conditions aids to maintain vascular homeostasis, by means of the vasodilating effects of insulin, during temporary stresses.

Whichever the mechanism involved, partially or totally eNOS-deficient mice had normal glycemia and increased circulating levels of insulin, although to a different extent. Interestingly, eNOS^+/−^ mice showed an approximate 40% increase in body weight (from 4 to 16 weeks of diet) that was significantly higher than in WT and comparable to that of HFD-fed mice. It is conceivable that the presence of even a slight hyperisulinemic state early in life in these mice, not associated with a true insulin resistance, would probably induce increased weight gain. This pattern might not apply to eNOS^−/−^ mice, as the total deletion of the enzyme would likely induce multiple additional effects limiting body growth.

### Cardiac molecular changes and pathways downstream insulin

The cardiac molecular analysis of eNOS protein showed a roughly 50% reduction of the enzyme in eNOS^+/−^ mice and its absence in eNOS^−/−^ animals, as expected from the experimental model used. HFD did not influence the expression of this protein or its activation, as the levels of protein and its phosphorylated forms were balanced in all groups of animals. As a consequence, the ratio between peNOS/eNOS was similar in all groups. These results suggest that the link between genetic eNOS abnormalities and increased coronary vascular tone is related to the decreased levels of eNOS protein caused by the genetic background rather than to its phosphorylation/activation.

Insulin binding to its receptor triggers a phosphorylation cascade that on the one hand activates eNOS through PI3K/Akt pathway and on the other hand activates the pro-atherogenic MAPK/ERK1-2 pathway which is associated with mitogenesis, cell growth and the production of ET-1 [26], [27]. Accordingly, insulin may have a vasodilating effect through stimulation of NO production and a vasoconstrictive effect through stimulation of synthesis and release of ET-1 [15].

In our study, there was no significant difference in Akt activation (pAkt) among the experimental groups. Since pAkt is recognized as a major protein involved in eNOS phosphorylation [28], our finding is consistent with the documented normal ratio peNOS/eNOS. Conversely, in all eNOS-genetically modified groups there was a clear trend towards activation of ERK1-2 (as documented by higher pERK/ERK1-2 ratio as compared with the WT group). As a consequence, in these animals, the ratio between ERK1-2 and Akt was unbalanced towards the vasoconstrictive pathway. Our data confirm and expand previous evidence [20], [21], [29]–[32] that a primary genetically determined eNOS deficiency directly impairs endothelial function, possibly resulting in increased coronary resistance, and decreases coronary vasodilating capability. In addition we demonstrated that this condition was associated with an increase in ERK1-2 phosphorylation. Further studies are required to elucidate the specific signaling pathways involved. It is conceivable that the association of an altered eNOS expression with the imbalance of the downstream insulin-dependent molecular signals could be further detrimental to coronary function. In fact, together with lower NO bioavailability, the altered signal transduction pathways could cause increased production of ET1. Moreover, since circulating insulin increases as a side effect of eNOS deficiency, its interaction with insulin receptor would further activate a pro-vasoconstrictive environment. HFD did not affect the balance of cardiac molecular pathways downstream insulin receptor, consistently with the lack of effects on coronary tone. Previous experimental studies showed that HFD may actually affect the Akt/eNOS signal in peripheral arteries. For instance, in HFD-fed mice, a FFA-mediated impairment of both basal and insulin-induced eNOS phosphorylation has been reported in peripheral vessels [1] and associated with endothelial dysfunction and hypertension. However, different results have been obtained in coronary arteries of diet-induced obese rats, as the endothelial NO vasorelaxation in response to insulin and the underlying PI3K/Akt/eNOS pathway were preserved [33] even though the pERK/ERK/ET-1 pathway seemed activated. The differential effects of HFD on insulin molecular pathways in peripheral arteries as compared with coronary vessels and cardiac tissue might explain the negligible effect of diet on coronary vascular resistance documented in the present study.

Together with eNOS, the cardiac expression of the inducible isoform of the enzyme was measured. In previous studies we have reported an increased expression of iNOS in cardiac tissue in animal models of metabolic impairment and secondary reduction of eNOS expression [18], [22], [23]. Decreased levels of eNOS and augmented expression of iNOS, together with enhanced oxidative stress, have been implicated in the pathogenesis of abnormal coronary function and potential myocardial damage [34]. The stress-induced iNOS isoform is able to produce an abnormal amount of NO, which, in an oxidative environment, reacts with superoxide, producing peroxynitrite and thus losing its vasodilating properties. Furthermore, peroxynitrite causes vasoconstriction and nitrosylation of cardiac proteins potentially resulting in functional coronary and myocardial damage. Accordingly, we have previously shown that a pharmacological treatment able to restore eNOS/iNOS ratio and counteract oxidative stress resulted in an increase of NO bioavailability associated to improvement of coronary vascular tone and myocardial function [18], [22], [23]. In the present study, iNOS expression in cardiac tissue was elevated in all eNOS deficient mice. Whether this pattern could further contribute to the abnormal coronary function and could potentially be reversed by treatment is an interesting matter of future investigation.

### Clinical implications and conclusion

While there is no evidence on genetic variants leading to a total eNOS deficiency in humans, some polymorphic variants of the gene encoding eNOS, such as Glu298Asp and −786 T/C SNPs, have been shown to alter the function or the expression of the enzyme [12], [35]. These variants have been associated with coronary disease, hypertension, insulin resistance and obesity in clinical populations [11]–[13], [36]–[40]. In this study partial eNOS knockout mice have been used to mimic this clinical condition of reduced eNOS expression and to study the interaction with HFD, while eNOS total knockout mice represented a reference extreme condition (not found in the clinical setting) with more marked abnormalities in all the parameters related with eNOS complete deficiency. Altogether, the results of the present experimental study, although not immediately translatable to humans, strongly highlight a role of eNOS gene both as a key regulator of, and a link between metabolism and coronary function. These findings could be clinically relevant, taking into account that in human populations the prevalence of eNOS polymorphism ranges from 5 to 35% [41]–[46]. It is intriguing to speculate that known or still unknown genetic abnormalities of the eNOS gene can be a common substrate of major abnormalities predisposing to coronary disease.
